# Prediction of adverse drug reactions based on knowledge graph embedding

**DOI:** 10.1186/s12911-021-01402-3

**Published:** 2021-02-04

**Authors:** Fei Zhang, Bo Sun, Xiaolin Diao, Wei Zhao, Ting Shu

**Affiliations:** 1 Department of Information Center, Fuwai Hospital, National Center for Cardiovascular Diseases, Chinese Academy of Medical Sciences and Peking Union Medical College, No. 167 North Lishi Road, Xicheng District, Beijing, 100037 China; 2 National Institute of Hospital Administration, National Health Commission, Building 3, Yard 6, Shouti South Road, Haidian, Beijing, 100044 China

**Keywords:** Adverse Drug Reactions, Knowledge Graph Embedding, Word2Vec, DrugBank

## Abstract

**Background:**

Adverse drug reactions (ADRs) are an important concern in the medication process and can pose a substantial economic burden for patients and hospitals. Because of the limitations of clinical trials, it is difficult to identify all possible ADRs of a drug before it is marketed. We developed a new model based on data mining technology to predict potential ADRs based on available drug data.

**Method:**

Based on the Word2Vec model in Nature Language Processing, we propose a new knowledge graph embedding method that embeds drugs and ADRs into their respective vectors and builds a logistic regression classification model to predict whether a given drug will have ADRs.

**Result:**

First, a new knowledge graph embedding method was proposed, and comparison with similar studies showed that our model not only had high prediction accuracy but also was simpler in model structure. In our experiments, the AUC of the classification model reached a maximum of 0.87, and the mean AUC was 0.863.

**Conclusion:**

In this paper, we introduce a new method to embed knowledge graph to vectorize drugs and ADRs, then use a logistic regression classification model to predict whether there is a causal relationship between them. The experiment showed that the use of knowledge graph embedding can effectively encode drugs and ADRs. And the proposed ADRs prediction system is also very effective.

## Background

Adverse drug reactions (ADRs) refer to undesired reactions during normal medication use [1], and they contribute to more than 20% of clinical trial failures and are considered a major burden in the modern drug discovery process [2, 3]. Serious ADRs can cause severe disability and even death in patients. In Europe, approximately 3.6% of all hospital admissions are caused by ADRs, and up to 10% of patients in European hospitals experience an ADR [3]. In the United States, it has been estimated that more than 2 million severe ADRs occur in hospitalized patients each year, resulting in more than 100,000 deaths [4, 5]. The annual financial cost of drug-related morbidity in the United States (US) was estimated at $528.4 billion in 2016, equivalent to 16% of total US healthcare expenditures that year [6].

Drugs are tested on animals and large human cohorts before clinical application to identify possible ADRs; however, because of limited sample size and duration of premarket trials, lack of heterogeneity of trial subjects, and numerous potential side effects and drug combinations, many adverse reactions may not be detected in the early stages of drug development [7]. ADRs therefore pose a significant risk to patient health and healthcare costs, and they are considered a major global public health issue. Researchers have explored multiple methods to predict individual drugs and combinations of drugs that may result in ADRs. Modern computer technology has aided this work, with methods such as machine learning being used to accelerate the prediction process and reduce the cost of drug development [8].

### Machine learning for ADR prediction

The method based on knowledge base (KB) has great advantages in accuracy and interpretability, but it needs a large number of clinical trials to collect the related adverse drug reaction events and construct the adverse drug reaction database. It is impossible to foresee the adverse reactions not shown at present. Machine learning related methods can be used to predict the potential adverse events that do not appear in the adverse reaction database. There is a large body of research on ADR prediction using machine learning methods. For example, Perez Nueno et al. [9] used canonical correlation analysis to predict the possible ADRs of drugs based on their physico-chemical properties and target protein information. Dey, et al. [10] used convolutional neural networks to extract chemical characteristics of drugs, encode different substructures of the drugs into feature vectors of the same length, and train a logistic regression classifier for each ADR. Acknowledging the importance of information on protein-protein interactions and drug-drug interactions, Hu, et al. [11] integrated these interactions into the distributed expression of drugs through a stacked deep heterogeneous network and trained an encoder for each semantic type. For each drug, the output of all the encoders were stitched together and used as the input of the second embedding model. The fully connected layer was then used for ADR prediction. Luo, et al. [12] used AutoDock Tools 1.5.6 and AutoDock Vina 1.1.2 software to dock drug molecules to each of the protein structures and used information on the drug’s substructure to vectorize the drug. The authors then trained a logistic regression classifier for each ADR.

Prior studies have been similar in their construction of ADR classifiers and use of traditional machine learning classification models. The key difference is how the drugs and ADRs were vectorized. In the aforementioned studies, the authors used only the information of a single drug when extracting the characteristics[10–12]. Using this approach, the associations between the drugs and other entities are not directly integrated into the vectors, and useful information may be lost. The knowledge graph (KG) and its embedding process have emerged in recent years as a helpful tool to not only represent the rich relationships between entities but also to directly encode these complex relationships into vectors. Using KG embedding to vectorize drugs and other entities is there for expected to better characterize a drug and other nodes.

Bean, et al. [13] constructed a KG containing four nodes (drug, indication, ADR, target), used the neighboring matrix of the drug nodes for its vectorization, and designed a classifier similar to the logistic regression classifier to predict ADRs. Munoz, et al. [14] also used KG to unify heterogeneous data from multiple databases. They treated ADR prediction as a multi-label classification problem, comparing multiple classification models on different datasets. In these prior studies using KG methodology, a predictive model was built for each ADR. In the present study, however, we combined ADR prediction tasks with KG embedding to predict potential adverse reactions of marketed drugs through a unified predictive model.

**Fig. 1:**
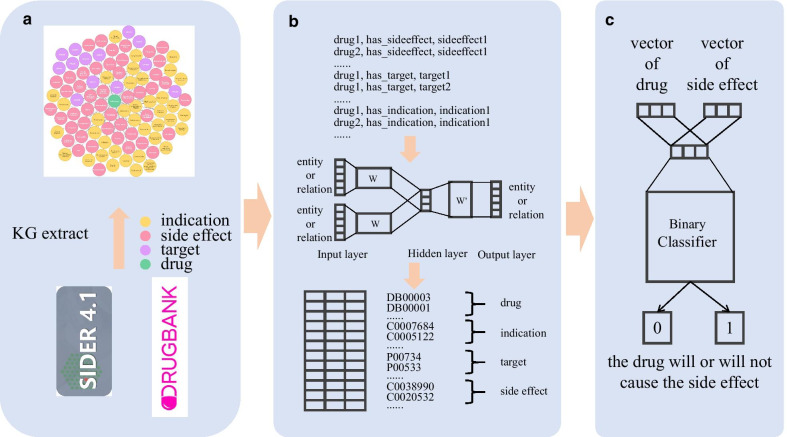
Overview of the work flow of this study. **a** Knowledge graph composed of the drug, targets, indications, and side effects extracted from the DrugBank and SIDER databases; **b** The knowledge graph embedding process, (b-top) Word2Vec training corpus constructed based on the knowledge graph; (b-middle) Continuous bag-of-words (CBOW) implementation process of Word2Vec, where the input layer inputs any two elements in the triple, the other element is used as the output (represented by one-hot vector), and *W* is the vector matrix of the training elements (entities and relations); (b-bottom) vector matrix of the training elements, *W*; and **c** Binaryclassifier, with the vector difference of the drug and side effect pair as the input and the probability that the drug may cause the side effect as the output

Our work flow is shown in Fig. 1. First, we constructed a KG containing four types of nodes (drug, indication, target, side effect) (ADRs were labeled as side effects in the database used for our work) and developed a new KG embedding method to embed the complex relationships between drugs, indications, targets, and side effects in the KG into a multidimensional vector. We then constructed a classification model for vectorized drugs and side effects to predict ADRs. Finally, we used liver injury as an example to predict the probability of drug-induced liver injury for all the drugs incorporated in the KG. For drugs with a higher probability of the ADR according to our model, we conducted a literature search to confirm our prediction.

## Methods

### Databases and KG construction

We constructed a KG with four types of nodes (drug, side effect, target, indication) and three relationships (has side effect, has target, has indication). The side effects, targets, and indications were regarded as characteristics of the drugs.

The drugs and their corresponding targets and anatomical therapeutic chemical (ATC) codes were extracted from the DrugBank database (version 5.1.4) [15], which is an open and free drug database that provides a variety of information on drugs (e.g. target, chemical properties, pharmacology, toxicology) and is often used in drug discovery and ADR prediction research. The database includes 13,450 drugs, including 2616 approved small molecule drugs, 1349 approved biopharmaceuticals (proteins, peptides, vaccines, and allergenics), 130 healthcare drugs, and more than 6340 experimental drugs. Drugs are represented in the database by their unique code, Drugbank_ID, and targets are represented by their unique code from the Uniprot database, uniprot_ID.

Drug indications and side effects were extracted from the SIDER database (version 4.1) [16] using the ATC code. The data included in SIDER are mainly extracted from drug manuals and are coded according to the Medical Dictionary for Regulatory Affairs, which is a clinically validated standard medical terminology dictionary that is often used to report adverse drug events. The current version of the SIDER database contains 5868 adverse reactions to 1430 drugs, with 139,756 *drug-side effect* pairs.

We collected information on 3632 drugs (only drugs with at least one characteristic were included), 2598 indications, 4286 targets, 5589 side effects, and 154,239 relationships among the different entities. We constructed and visualized the KG using Neo4j, as shown in Fig. 1a. The number of entities in the KG and their relationships with the drugs are shown in Table 1.

**Table 1:** Number of entities and relationships in the knowledge graph

	Drug	Side effect	Target	Indication	Total
Entities	3632	5589	4286	2598	12473
Drug	–	126791	13851	13597	154239

### KG embedding

**Fig. 2:**
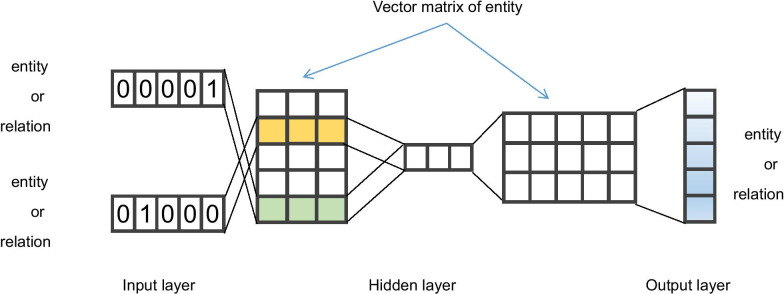
Overview of knowledge graph embedding

Word2Vec is a classic word embedding method in Natural Language Processing. Using this method, a model to vectorize words can solve the problem of sparseness brought by atomic methods such as bag of words as well as embed the context information of words in sentences into word vectors [17]. Word2Vec can be implemented through the continuous bag-of-words (CBOW) and Skip-gram architectures. CBOW uses the context words of the center word to predict itself, and it is suitable when the dataset is small. Skip-gram is used to predict the context words of the center word, and it is generally applied to large datasets [17]. Rather than using Word2Vec for the prediction model itself, we used it to obtain the matrix of word vectors generated during model training. Because the word vector contains the context information of the word, it is widely used in semantic analysis. In the KG, a triple is exactly a *subject*-*predicate*-*object* sentence. In the context of ADRs, a triple *(drug 1, has side effect, side effect 1)* indicates that drug 1 has the side effect 1. Therefore, if the KG is considered as a corpus composed of triples and the two elements in the triple are considered as the context of the third element, the Word2Vec model can be used to vectorize the graph and simultaneously embed the head and tail entities and relationship in the triple into the vector. The work flow of the KG embedding process is shown in Fig. 2.

The KG embedding model is expressed by functions (1), (2), and (3):

1 $$\begin{aligned}&f(x_{i}^{1},x_{i}^{2})=softmax \left( (x_{i}^{1}+x_{i}^{2})\cdot W_{1}\cdot W_{2}\right) \end{aligned}$$

2 $$\begin{aligned}&p(x_{i}^{3}|x_{i}^{2},x_{i}^{1}) = f(x_{i}^{1},x_{i}^{2})\cdot x_{i}^{3} \end{aligned}$$

3 $$\begin{aligned}&Loss=-\sum _{i}log\left( p(x_{i}^{3}|x_{i}^{2},x_{i}^{1}) \right) \end{aligned}$$

In the functions, $x_{i}^{2}, x_{i}^{1}$ are the $i-th$ sample of the model input, which are the one-hot vectors of any two elements in the triple; the dimensions of the vectors are both $1 \cdot n$, $W_{1}$and $W_{2}$ are the final required entity vector matrices. The matrix dimensions are $n \cdot size$ and $size \cdot n$, randomly initialized. One vector matrix is selected as the entities’ vector (each row corresponds to an entity as shown in Fig. 1b). In the KG, *n* is the total number of entities and relations (after removing duplicates), and *size* is the dimension of the entity vector desired. Function (1) represents the probability of each entity as the output when the elements of the triple are used as input, and as function (2), the product is multiplied by the one-hot vector of the remaining entity in the triple to obtain the probability of the third element in the triple. The training target is to maximize the probability, which involves minimizing the loss function (3). Using this method, all nodes and relationships in the KG can be embedded into a vector.

### Prediction model

Predicting whether a certain drug will produce an adverse reaction involves predicting whether there is a *has side effect* relationship between the two entities, which is equivalent to performing KG completion. Therefore, the ADR prediction problem can be converted into a binary classification problem to judge whether there is a *has side effect* relationship between the drug and ADR.

We used logistic regression to implement the binary classification model for ADRs as shown in Fig. 1c. Logistic regression is the most basic and important method in the classification algorithm, and it provides a model that is simple and easy to implement. The difference between the vector of $drug_{i}$ and $ADR_{j}$ is used as the model input *x*, and whether there is a *has side effect* relationship between them is used as the output (1=yes, 0=no like function (4)) to train the model, the details of the training data for the classifier are described in section *Datasets*. The probability of a $drug_{i}$ causing an $ADR_{j}$ is calculated as function (5):

4 $$\begin{aligned}&y_{i}(j)=\left\{ \begin{array}{ll} 1&{}if\,drug\,i\,causes\,ADR\,j\\ 0&{}other \end{array}\right. \end{aligned}$$

5 $$\begin{aligned}&\bar{y_{i}}\left( j\right) =P\left( Y=1|x;w,b\right) =\frac{1}{1+exp\left( w\cdot \left( x_{ADR_{j}}-x_{drug_{i}}\right) +b\right) } \end{aligned}$$

The model parameters *w* and *b* are obtained by minimizing the loss *J* in function (6) using the training dataset, where $y_{i}$ represents the standard output (0 or 1) of the $i-th$ sample and $\bar{y_{i}}$ represents the output of the model when the input is $x_{i}$ (the difference between the vector of drug and ADR in $i-th$ sample), *m* represents the total number of samples.

6 $$\begin{aligned} J(w,b)=-\frac{1}{m}\sum _{i=1}^{m}\Big [y_{i}log\left( \bar{y_{i}}\right) +\left( 1-y_{i}\right) log\left( 1-\bar{y_{i}}\right) \Big ] \end{aligned}$$

### Model evaluation

We evaluated our model based on its prediction performance on the test datasets and by comparison with the literature on ADR prediction. Specifically, the area under the receiver operating characteristic curve (AUC) was used to evaluate the model classification effect. The receiver operating characteristic (ROC) curve is obtained by using the false positive rate (FPR) and the true positive rate (TPR) corresponding to the classifier under different classification thresholds. AUC is determined by the area enclosed by the ROC curve and the FPR axis, with a higher AUC indicating better discrimination. The TPR and FPR are calculated as follows:

7 $$\begin{aligned} \begin{array}{ll} FPR=\frac{FP}{FP+TN}\\ TPR=\frac{TP}{TP+FN} \end{array} \end{aligned}$$

where *FP* indicates the number of incorrect predictions in the positive samples, *TN* indicates the number of correct identifications in the negative samples, *TP* indicates the number of correct predictions in the positive samples, and *FN* indicates the number of incorrect identifications in the negative samples. We also calculated the *recall*, *precision*, and $F-score$ for the model to evaluate its classification performance:

$$\begin{aligned} \begin{array}{ccl} Precision&{}=&{}\frac{TP}{TP+FP}\\ Recall&{}=&{}\frac{TP}{TP+FN}\\ F-score&{}=&{}\frac{2*Precision*Recall}{Precision+Recall} \end{array} \end{aligned}$$

## Results

### Datasets

There were 3632 drugs and 4286 targets extracted from the DrugBank database that were matched by ATC code to 5589 types of side effects and 2598 types of indications in SIDER (Table 1). Using the extracted data, we noted that the side effects of some drugs are the indications of other drugs. Because the indication of a drug cannot be the side effect of itself, the *drug-indication* pairs can be regarded as a negative sample of the classifier, with the corresponding tag *has no side effect* labeled as 0. To ensure the maximum amount of indication information could be embedded in the drug vector during the KG embedding and classifier training phase, we randomly selected only 10% (1359) of the 13,597 *drug-indication* pairs to serve as the negative sample in the test dataset. An equal number of the *drug-side effect* pairs were randomly selected as the positive sample of the test dataset. These two sets of data were reserved for model testing and were not included in the training of the KG embedding model and the classifier, simulating the potential unknown ADRs that may occur in real-world practice.

The remaining triples *(drug, has target, target)*, *(drug, has indication, indication)*, and *(drug, has side effect, side effect)* together formed the corpus to train the KG embedding model; the *(drug, has indication, indication)*(copied 10 times to avoid sample imbalance) and *(drug, has side effect, side effect)* were then used to train the ADR prediction classifier. The details of the final data split are shown in Table 2.

**Table 2:** Data used for knowledge graph embedding and adverse drug reaction classifier training and testing

Triple	Knowledge graph embedding	Classifier training	Classifier testing	Total
*(drug, has target, target)*	13851	0	0	13851
*(drug, has indication, indication)*	12238	12238*10$^\Delta$	1359	13597
*(drug, has side effect, side effect)*	125432	125432	1359	126791

$^\Delta$ To avoid sample imbalance, the drug-indication pairs used for the training of the ADR classification model were replicated and expanded 10 times

### Evaluation of KG embedding and parameters of the ADR prediction model

The most important parameters of the Word2Vec model are *iter* and *size*. *iter* refers to the number of iterations the Word2Vec model trained, and *size* is the entity vector dimension obtained by the model. In general, the more iterations the KG embedding model trains, the better the vector will fit to the KG. Accordingly, we assessed whether the KG embedding process helped to encode information on entities such as drugs and side effects into vectors by evaluating the prediction performance of the ADR classifier under different iterations of the KG embedding model.

#### Parameter settings

When using the Word2Vec model to implement the KG embedding, we set $min\_count$ to 1 (indicating that the nodes that appeared less than once in the corpus should be deleted) to ensure all nodes could be vectorized. Because a sentence consists of three elements of a triple, the maximum sentence length was 3, the *window* set to 2 and the implementation *method* set to CBOW. We used the Python Gensim package (3.8.1) to implement KG embedding.

The ADR prediction model used logistic regression, with the default *L*2 regularization term, the default *C* value of 1, and the loss function optimization algorithm set to stochastic average gradient (SAG; this sets the parameter *solver* to SAG). To ensure model convergence, the maximum number of iterations was set to 10,000. The classifier model was implemented using the Python sklearn package (0.21.3).

#### Analyses and results

We used the training and testing data split described in section to train and evaluate our ADR prediction model under different iterations of KG embedding. Because our goal was to determine whether the KG embedding process helped to encode information on entities, a separate verification dataset was not needed, and the test set was used to both evaluate the model and directly identify the best parameters.

**Fig. 3:**
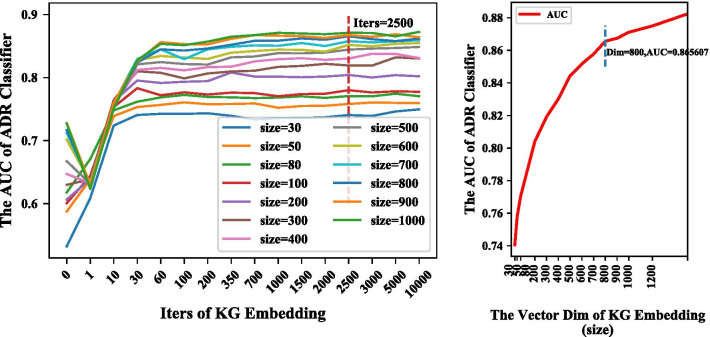
Parameter select. Area under the receiver operating characteristic curve (AUC) of the adverse drug reaction (ADR) classifier in the test set under different vector sizes and iterations of knowledge graph (KG) embedding (left), and AUC of the ADR classifier in the test set under different vector dimensions when iterations = 2500 (right). Dim indicates dimension; iters, iterations

For all sizes of the entity vector, the AUC of the ADR prediction model increased as the number of iterations of KG embedding increased (Fig. 3, left). The increase in prediction performance gradually slowed after 60 iterations, indicating that the KG information could not be fully integrated into the vector when the number of iterations was too low. As the iterations increased, the vector was better able to represent each entity. Based on these results, the KG embedding process was able to sufficiently encode the graph information into the entity vector.

The classifier’s performance also increased as the vector size increased (Fig. 3, right). For the curves with high AUC, performance was maximized at 2500 iterations of KG embedding. Figure 3(right) shows the classification performance for different vector sizes at 2500 iterations. The increase in AUC slowed after the vector size reached 500 and 800. Based on the KG embedding time, ADR classifier training time, and the ADR classifier performance, $iter = 2500$ and $size = 800$ were selected as the optimal parameters for the prediction model.

### Evaluation of ADR prediction model

We evaluated the ADR prediction model by identifying the *drug-side effect* and *drug-indication* pairs in the testing dataset and comparing the AUC for the prediction model with results from similar research in the literature. To increase the credibility of the model evaluation, we shuffled the original data set and randomly divided it into new training and testing sets according to the method described in section *Datasets*. The KG embedding and ADR classifier training were repeated from scratch using the new training set, and the model performance was evaluated using the new test set. As determined in section *Analyses and results*, the number of iterations of KG embedding was set to 2500, the vector dimension to 800, the window size to 2, min_count to 1, and the implementation method to CBOW. We repeated this process six times to assess the stability of the results.

#### Results

**Fig. 4:**
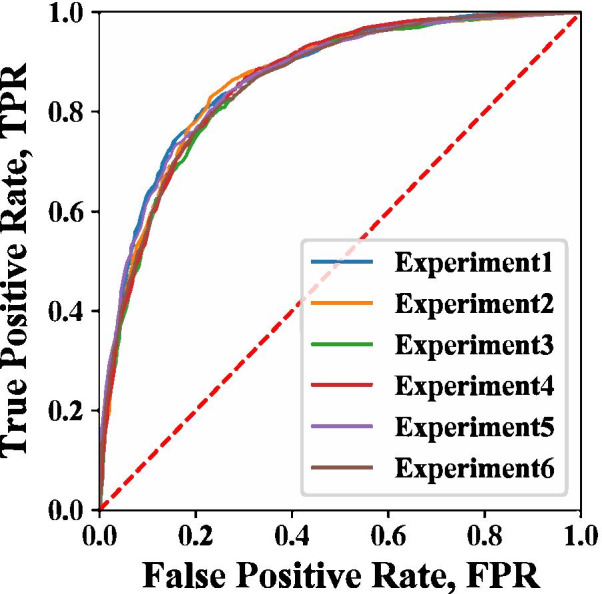
Receiver operating characteristic curves for the six experiments

In the testing dataset containing 1359 positive samples and 1359 negative samples, the AUC of the classification model reached a maximum of 0.870, and the mean AUC was 0.863 (Table 3; Fig. 4). The *precision*, *recall*, and $F-score$ are shown in Table 3. The ROC curve and other evaluation indicators were stable across the six repeated experiments without obvious fluctuations, indicating a stable prediction model.

**Table 3:** Evaluation results of the adverse drug reaction prediction model on the test set

	Precision	Recall	F-score	AUC
Experiment 1	0.779	0.819	0.799	0.87
Experiment 2	0.79	0.81	0.8	0.863
Experiment 3	0.775	0.799	0.787	0.855
Experiment 4	0.773	0.819	0.796	0.862
Experiment 5	0.779	0.804	0.791	0.867
Experiment 6	0.77	0.812	0.79	0.86
Mean	0.778	0.81	0.794	0.863

We compared our model with eight related ADR prediction studies (Table 4) in two ways, average AUC over all ADRs and AUC on top 10 ADRs prediction models. The AUC of our model are higher than that reported for most of the ADR models in the literature, indicating that our model had better prediction performance. Besides, most prior prediction models were trained separately for each ADR, resulting in the training of a large number of models. In contrast, we first encoded the drug and side effect information into their own vectors and subsequently scored drug and side effect pairs directly through a unified logistic regression model to determine whether there was a relationship between the two entities, greatly reducing the number of models. One previous prediction model [13] also used the KG approach and reported a high AUC; however, this model included only a few specific ADRs and may not result in high AUC values for other ADRs. Additionally, the model only used the number of targets in the characterization process for a drug and did not consider the specific content of the targets, resulting in the loss of potentially useful information.

**Table 4:** Comparison of adverse drug prediction models in the literature

Study	Drug	Features	ADRs	Label Source	AUC all	AUC top10
Our work	3632	Drug targets, indications, and ADRs	5589	SIDER	0.86	0.934
Luo et al.[12]	655	Molecular docking (600 proteins)	1533	SIDER	0.84	–
LaBute et al.[24]	560	Molecular docking (409 proteins)	85 (10 groups)	SIDER	0.60–0.69	–
Bean et al.[13]	524	Drug targets, indications, and ADRs	10	SIDER/EHR	–	0.92
Cao et al.[25]	746	Structures, gene expressions, and multiple evidences sources	817	SIDER	0.57-0.88	–
Jamal et al.[26]	928	Chemical, biological, and phenotypic properties	22	SIDER	0.48–1.00	–
Hu et al.[11]	548	DDI, PPI, drug target and treatment information, chemical structures, and side effects	1318	SIDER	0.84	–
Dey et al.[10]	1430	Structure information and side effects	1766	SIDER	–	0.919

A portion of the data were collected from Luo et al. [12];

ADR, adverse drug reaction; AUC, area under the receiver operating characteristic curve;

DDI, drug-drug interaction; PPI, protein-protein interaction;

EHR, Electronic Health Records

### Literature evidence

To further verify the feasibility of our prediction model, we collected reports on ADRs from the literature. We used our prediction model to score these drug-ADRs, which were not included in our KG. Using liver injury-related ADRs as an example, we tested our model’s predictive ability in two ways.

In the first approach, we searched the liver injury-related literature in PubMed. Ten of the first 20 articles reported on liver injury caused by drugs. Two papers reported on related drugs that did not appear in our KG [18, 19], so we were unable to predict the ADRs for these drugs. We used our model to score the liver injury-related ADRs for the drugs in the remaining seven articles. The studies, ADRs reported, and prediction results from our model are shown in Table 5.

**Table 5:** Adverse drug reactions obtained from PubMed and probability of their occurrence according to our model

Study	Drug	Adverse Drug Reaction	Probability
Kuniyosh et al. [27]	Atorvastatin (DB01076)	Liver injury (C0160390)	0.955
Brehm et al. [28]	Acetaminophen (DBDB00316)	Acute liver injury (C2242583)	0.62
Moon et al. [29]	Albendazole (DB00518)	Liver injury (C0160390)	0.919
Kopecky et al. [30]	Nivolumab (DB09035)	Liver injury (C0160390)	0.854
Gisi et al. [31]	Azathioprine (DB00993)	Hepatitis cholestatic (C0149904)	0.958
Carretero et al. [32]	Osimertinib (DB09330; one of the tyrosine-kinase inhibitors)	Hepatotoxicity (C0235378)	0.797
Ota et al. [33]	Crizotinib (DB08865)	Liver injury (C0160390)	0.83
Kawaguchi et al. [18]	Laninamivir octanoate (DB11888)	Liver injury (C0160390)	Drug not in KG
Kwan et al. [19]	Pembrolizumab (DB09037)	Hepatotoxicity (C0235378)	Drug not in KG
Rajan et al. [34]	Sevoflurane anaesthesia (DB01236)	Hepatotoxicity (C0235378)	0.959

KG, knowledge graph;

The code in brackets after the drug is its Drugbank_ID;

The code in brackets after the Adverse Drug Reaction is its UMLS ConceptID

In the second approach, we calculated the probability of liver injury (UMLS ConceptID: C0160390) for all drugs in the KG and arranged them in descending order of probability. We searched the literature to verify the 10 drugs with the highest probability for liver injury as an ADR. No published studies were identified for two of the drugs, and the literature indicated that two drugs did not cause liver injury. One drug was shown to cause liver damage when combined with other drugs (montelukast sodium) [20]. One drug did not harm the liver with normal use, but long-term use was associated with pathological changes in the liver, including liver injury and liver fibrosis [21]. There was clear evidence of the remaining four drugs causing liver damage. The prediction results from our model are shown in Table 6.

**Table 6:** Top 10 drugs predicted to cause liver injury according to our model

Drug	Cause of Liver Injury According to Literature	Study
Valganciclovir	No	Ganciclovir [35]
Reboxetine	–	No literature
Argatroban	No	Levine et al. [36]
Tibolone	Yes	Macedo et al. [37]
Dextroamphetamine	Yes	Vanga et al. [38]
Trovafloxacin	Yes	Giustarin et al. [39]
Tamsulosin	Yes	Fremond et al. [20]
Iopromide	Yes	Bolado Concejo et al. [40]
Naltrexone	Yes (long-term use)	Zheng [21]
Frovatriptan	–	No literature

### Case study

In order to connect our model with the occurrences of the possible ADRs of some drugs in the real, we collected drugs from DILIrank dataset, and predicted the probability they cause the Drug-induced liver injury (DILI, UMLS ConceptID: C0860207). DILIrank consists of 1,036 FDA-approved drugs and divided into four classes according to their potential for causing DILI [22]. DILI classification is based on the analysis of hepatotoxicity descriptions in FDA-approved drug labeling documents and causal evidence in the evaluation literature. Specifically, this largest publicly annotated DILI dataset consists of three groups (Most-, Less- and No-DILI concern) with strong causal evidence that drugs are associated with liver injury, while the causal relationship of another group (Ambiguous-DILI-concern) is unknown.

The average prediction probabilities of 862 drugs which can be found in KG and DILIrank were calculated (Table 7). The results show that our model has good discrimination in predicting whether the drug will lead to DILI or not, but it can not predict the severity. That is due to the original data, which only contains the information about whether the drug will cause ADRs or not, but without the severity. It is worth noting that the prediction probability of “Ambiguous-DILI-concern drug” category is 0.578, which is higher than that of “No-DILI-concern drug” (0.470). The results accord with the real situation of the DILIrank dataset, because the drugs that may lead to DILI are in the group of “Ambiguous-DILI-concern drug”.

**Table 7:** Number of drugs we studied and corresponding average probabilities from our model

DILI concern	Drug in KG	Average probalility
Most-DILI-concern drug	141	0.573
Less-DILI-concern drug	257	0.607
No-DILI-concern drug	243	0.470
Ambiguous-DILI-concern drug	221	0.578

## Discussion

Drug safety is an important component of medical care and the process of drug development. Because it is not possible to test all combinations of drugs by screening of ADRs through experiments and clinical trials [7], data mining technology has emerged as a promising approach to predict drugs that result in ADRs. This approach can both guide the drug development process as well as provide a reference for doctors when prescribing.

Many studies have used computer-aided detection of ADRs. These studies have generally followed the same steps [23]:

- Vectorize the drug
- Train an ADR classifier based on the drug’s vector

Traditionally, vectorization methods have characterized drugs by their surrounding properties, such as the chemical structure and target, and vectorization of each drug was performed separately. The connection between drugs is not generally considered, potentially resulting in loss of information. The KG approach provides an effective means to represent the correlation between data. When a drug and its surrounding attributes are represented by a KG, drugs can be connected through a common structure or property, and attributes can be connected through a common drug. When embedding the KG (vectorizing the nodes in the graph), the complex relationships between the nodes can be embedded into the vector at the same time, resulting in a drug vector with more information.

Our experimental results showed that an increase in the degree of KG embedding increased the model prediction performance, indicating that the process of KG embedding can effectively embed information into the vector. While vectorizing drugs, the ADR and target were also vectorized. Therefore, we may also be able to replicate the process for the prediction of drug targets and drug indications. By classifying the difference between drug and ADR vectors, a unified prediction model can be obtained without having to build individual prediction models for each ADR, greatly increasing the versatility of the model.

## Conclusion

In this paper, we introduce a new knowledge graph embedding method to represent drugs and ADRs, then use a logistic regression classification model to predict whether there is a causal relationship between them. The experiment showed that the use of knowledge graph embedding can effectively encode drugs and ADRs. And the proposed ADRs prediction system is also very effective. We believe combined with knowledge graph, the information of drugs, ADRs and target proteins can be better represented, which is of great significance for the study of ADRs prediction. In future research, we will add the structure information of drugs and protein target to the knowledge graph, and use the longer path in the knowledge graph as the input of Word2Vec model, which may make the scope of information perceived by entities wider.

## Data Availability

The datasets supporting the conclusions of this article are available in the DrugBank [15](https://go.drugbank.com/releases/latest) and SIDER [16](http://sideeffects.embl.de/) repository. And The project will be released in https://github.com/zf-go/ADR after publication.

## Abbreviations

ADRs: Adverse drug reactions
KG: Knowledge graph
ATC: Anatomical therapeutic chemical
CBOW: Continuous bag-of-words
AUC: Receiver operating characteristic curve
ROC: Receiver operating characteristic
FPR: False positive rate
TPR: True positive rate
UMLS ConceptID: The ADR’s unique identification in United Medical Language System
DrugbankID: The drug’s unique identification in Drugbank database
